# Editorial: Molecular and Biotechnological Advancements in *Hypericum* Species

**DOI:** 10.3389/fpls.2016.01687

**Published:** 2016-11-11

**Authors:** Gregory Franklin, Ludger Beerhues, Eva Čellárová

**Affiliations:** ^1^Department of Integrative Plant Biology, Institute of Plant Genetics of the Polish Academy of SciencesPoznań, Poland; ^2^Institute of Pharmaceutical Biology, Technische Universität BraunschweigBraunschweig, Germany; ^3^Department of Genetics, Institute of Biology and Ecology, Faculty of Science, Pavol Jozef Šafárik UniversityKošice, Slovakia

**Keywords:** *Hypericum* spp., biosynthetic pathways, biotechnology, genomics, pharmacology

This special issue on the genus *Hypericum* (family *Hypericaceae*) consists of 12 articles focusing on recent advancements related to biosynthetic pathways, biotechnology, molecular biology, genomics, pharmacology, and related disciplines. *Hypericum* is well-known for its medicinal properties. There are about 487 *Hypericum* spp., which are distributed across every continent except Antarctica. Although the Mediterranean basin was recognized as a hot spot for *Hypericum* spp., Asian and American continents also account for significant biodiversity of *Hypericum* spp., out of which many are endemic.

Due to anthropogenic exploitation and unsustainable collection practice, several *Hypericum* spp. have become critically rare/endangered and at least 17 species are included in the International Union for Conservation of Nature red list. The review by Bruňáková and Čellárová deals with conservation strategies in the genus *Hypericum* via cryogenic treatment. The authors discuss the recent advances in the conventional two-step and vitrification-based cryopreservation techniques in relation to the recovery rate and biosynthetic capacity of *Hypericum* spp. Moreover, freezing tolerance as a necessary pre-condition for successful post-cryogenic recovery of *Hypericum* spp. is proposed.

Within the genus, *H. perforatum* is the most important species, which is used in the treatment of mild to moderate depression since ancient times. Oliveira et al. comprehensively review the neuroprotective properties of *H. perforatum* in terms of its main biologically active compounds, their chemistry, pharmacological activities, drug interactions, and adverse reactions. They also discuss how *H. perforatum* extracts and its major components protect neurons from toxic insults either directly or indirectly as antioxidants.

Hypericin is a characteristic constituent of the genus *Hypericum*, which can countervail complex diseases. Importantly, hypericin is a natural photosensitizing pigment and its photoexcitation properties are under intensive investigation with the aim of its utilization as a fluorescent diagnostic tool and anti-cancer agent for photodynamic therapy (PDT). Jendželovská et al. review the benefits of photoactivated and non-activated hypericin in preclinical and clinical applications focusing on multidrug resistance mechanisms.

The demand of the pharmaceutical industry for new active compounds and drug leads is the driving force behind phytochemical analysis of medicinal plants. Although *H. perforatum* is phytochemically well-characterized, several other species still need to be elucidated for their chemical profiles. Crockett et al. report the isolation of a new phloroglucinol derivative, 1-(6-hydroxy-2,4-dimethoxyphenyl)-2-methyl-1-propanone, from *H. cistifolium* and *H. galioides*. They also detect two new terpenoid derivatives in the later species. In addition to establishing the chemical structures of these new compounds using 2D-NMR spectroscopy and mass spectrometry, the *in vitro* antimicrobial and anti-inflammatory activities are analyzed.

In spite of many reports on the phytochemistry of *H. androsaemum*, the chemical composition of its red berries remained unknown. The study by Caprioli et al. reveals that a new tetraoxygenated-type xanthone is responsible for the red color of the berries. In addition, the authors observe high amounts of phenolic compounds in the red berries and show their cytotoxicity in human tumor cell lines.

Changes in the metabolome of *H. perforatum* root cultures in response to time of culture and chitosan treatment are reported by Brasili et al. For example, increases in biomass correlated with increases in phenolic compounds, such as xanthones including brasilixanthone B. Histological studies reveal that chitosan-treated roots undergo marked swelling of the root apex, which is mainly due to hypertrophy of the first two sub-epidermal layers and periclinal cell divisions.

Although hypericin is a major active compound of *Hypericum* spp., identified and characterized a century back, its biosynthesis is still not fully understood. *HYP1* was thought to be involved in the final stages of hypericin biosynthesis. There are two articles on *HYP1* in this issue. Karppinen et al. show that expression of *HYP1* genes is relatively high in leaves and increases after wounding and treatment with defense signaling compounds, such as salicylic and abscisic acids. HYP1 transcripts mainly occur in vascular tissues of root and stem and in leaves in mesophyll cells as well, as indicated by *in situ* hybridization.

Sliwiak et al. report the crystal structure of the HYP1 protein in complex with melatonin. This structure confirms the conserved protein fold and the presence of three unusual ligand-binding sites, two of which are in internal chambers, while the third one is formed as an invagination of the protein surface. Altogether, the studies of *HYP1* reveal that it may be involved, as a *PR10* gene, in plant defense responses, however, its role in hypericin biosynthesis is questioned.

Xanthones and flavonoids also contribute to the medicinal effects of *H. perforatum* extracts. Belkheir et al. analyze regulatory mechanisms underlying flavonoid and xanthone biosyntheses in *H. perforatum* using immunofluorescence localization and histochemical staining (Belkheir et al.). They observe that both chalcone synthase (CHS) and benzophenone synthase (BPS) are located in the mesophyll. However, CHS and BPS accumulate at different stages of leaf development, with CHS accumulation occurring earlier than that of BPS. Flavonoids were detected in the mesophyll, indicating that the sites of biosynthesis and accumulation coincide.

Transcriptome profiling is an unbiased approach for gene prediction. Using this tool, Velada et al. identify and characterize the alternative oxidase (AOX) protein family of *H. perforatum* during post-germination seedling development. Analysis of the intron regions of *AOX* reveals miRNA coding sequence polymorphisms with functional significance in regulation of gene expression at the posttranscriptional level. Moreover, the presence of a transposable element in the *AOX* intron region with still unidentified function is elucidated by *in silico* analysis.

Besides *H. perforatum, de novo* transcriptome profiling of four other *Hypericum* spp. namely, *H. annulatum, H. tomentosum, H. kalmianum*, and *H. androsaemum*, is reported by Soták et al. for the first time. The next-generation sequencing- acquired data provide a source of information for subsequent studies toward the search for candidate genes involved in the biosynthesis of hypericin. Comparative analysis of differentially expressed genes between hypericin-producing and hypericin-lacking species and tissues reveals more than 100 differentially upregulated contigs. These include new sequences with homology to octaketide synthase and enzymes that catalyze phenolic oxidative coupling reactions.

In spite of the recent advances in the understanding of biosynthesis-related gene expression in *H. perforatum*, functional genomics is still in its infancy, mainly due to its recalcitrance against *Agrobacterium tumefaciens* and low efficiencies of the reported transformation methods. Hou et al. propose a perspective on possible ways to achieve efficient transformation and hence improvements *via* metabolic engineering.

## Author contributions

All the authors contributed equally to the manuscript, and approved it for publication.

### Conflict of interest statement

The authors declare that the research was conducted in the absence of any commercial or financial relationships that could be construed as a potential conflict of interest.

